# Whole-transcriptome analyses of the Sapsaree, a Korean natural monument, before and after exercise-induced stress

**DOI:** 10.1186/s40781-016-0097-1

**Published:** 2016-04-15

**Authors:** Ji-Eun Kim, Junkyung Choe, Jeong Hee Lee, Woong Bom Kim, Whan Cho, Ji Hong Ha, Ki Jin Kwon, Kook Il Han, Sung-Hwan Jo

**Affiliations:** SEEDERS Inc, Daejeon, 34015 Republic of Korea; The Korean Sapsaree Foundation, Gyeongsan, Gyeongbuk 38412 Republic of Korea; Department of Animal training and event, Daekyeung University, Gyeongsan, Gyeongbuk 38547 Republic of Korea

**Keywords:** NGS, Bioinformatics, RNA-Seq, Transcriptome, Exercise, Physical stress, Sapsaree

## Abstract

**Background:**

The Sapsaree (*Canis familiaris*) is a Korean native dog that is very friendly, protective, and loyal to its owner, and is registered as a natural monument in Korea (number: 368). To investigate large-scale gene expression profiles and identify the genes related to exercise-induced stress in the Sapsaree, we performed whole-transcriptome RNA sequencing and analyzed gene expression patterns before and after exercise performance.

**Results:**

We identified 525 differentially expressed genes in ten dogs before and after exercise. Gene Ontology classification and KEGG pathway analysis revealed that the genes were mainly involved in metabolic processes, such as programmed cell death, protein metabolic process, phosphatidylinositol signaling system, and cation binding in cytoplasm. The ten Sapsarees could be divided into two groups based on the gene expression patterns before and after exercise. The two groups were significantly different in terms of their basic body type (*p ≤ 0.05*). Seven representative genes with significantly different expression patterns before and after exercise between the two groups were chosen and characterized.

**Conclusions:**

Body type had a significant effect on the patterns of differential gene expression induced by exercise. Whole-transcriptome sequencing is a useful method for investigating the biological characteristics of the Sapsaree and the large-scale genomic differences of canines in general.

**Electronic supplementary material:**

The online version of this article (doi:10.1186/s40781-016-0097-1) contains supplementary material, which is available to authorized users.

## Background

The Sapsaree (*Canis familiaris*) is a native Korean dog that is distributed throughout the Korean peninsula, and is very friendly, protective, and loyal to its owner. The Sapsaree population size decreased dramatically during the Korean War in the 1950s, and the breed was considered endangered. A program of systematic mating and reproduction to preserve the Sapsaree from extinction and maintain a pure pedigree generated the current population of about 4,000 individuals, including the 500 dogs now living at the Sapsaree Breeding Research Institute in Gyeongsan, Gyeongbuk province [1]. The Sapsaree was registered as a Korean Natural Monument (number: 368) in 1992. Although recent studies have shed light on the origin and various morphological and behavioral traits of the Sapsaree, the genetic backgrounds, abundant genetic polymorphisms, and novel genes of the Sapsaree are still not completely understood [2].

RNA sequencing (RNA-Seq) is one of the most useful next-generation sequencing tools for investigating the landscape of a whole transcriptome. Using RNA-Seq, researchers have identified differentially expressed and novel genes, unraveled the expression profiles underlying phenotypic changes, and discovered unannotated, transcriptionally active regions that cannot be detected by conventional gene prediction [3]. Furthermore, the recent use of RNA-Seq has captured the scale and complexity of organ-specific and tissue-specific transcriptomes, making RNA-Seq the technique of choice for investigating gene expression during complex phenomena such as stress [4].

Exercise is usually recognized as a stress factor, as is any environmental change that activates or pressures cells and tissues. And the level of response to physical exercise is different from individuals to individuals. So, it is needed to research the biological responses under physical stress. Several RNA-Seq studies of exercise-induced stress have been performed in equines [3, 4]. There has been little progress, however, in canine-based RNA-Seq studies, which have been limited to certain diseases. Therefore, we performed a large-scale analysis of whole-transcriptome data to investigate the gene expression levels before and after exercise in the Sapsaree. This study will provide the basic approach for identified the biologic characteristics and breeding the working dogs.

## Methods

### Morphological traits and blood sample collection

The dogs were handled in accordance with Article 23 ‘Experiments with Animal’ of Korea’s Animal Protection Law, 2015, and the Korean Sapsaree Foundation cooperated and approved all animal care procedures prior to the initiation of the experiment. Ten Sapsarees housed in controlled environmental conditions were used in the experiment. The basic morphological traits of each Sapsaree including body height (BH), body length (BL), depth of chest (DC), body weight (W), and hair color were measured before the experiment. Then, the dogs were exercised one-on-one by trainers for 1 h in 5 min intervals, with the trainers leading the dogs in quick step 10 times around a course on a 20 m × 20 m square field located at the Korean Sapsaree Foundation facility in Gyeongsan. The exercise course included hurdling (40 cm, 70 cm, and 80 cm), high jumping (50 cm), A-shape hurdling, bridge-shape hurdling, and seesaw hurdling.

Blood samples were drawn under a veterinarian’s supervision from a cephalic vein before and immediately after exercise, resulting in a total of 20 samples. Immediately after collection, portions of the blood samples were dispensed into serum separator tubes (SST) (1.5 ml in each tube [Greiner Bio One, Kremsmuenster, Austria]) to measure hormone levels, and into PAXgene blood RNA tubes (2.5 ml in each tube, [PreAnalytiX, Hombrechtikon, Switzerland]) for RNA sequencing according to the respective manufacturers’ protocols [5, 6]. The blood for serum was allowed to clot at room temperature before centrifugation. The serum was then separated and stored frozen at -70 °C until completion of the case enrollment and sample collection.

### Measurement the stress indicator in blood

Four substances related to physical stress were chosen based on previous studies: cortisol, aspartate aminotransferase (AST), creatine kinase (CK), and creatinine. The serum CK, AST, creatinine levels are commonly used to muscle damage indicators and renal function [7–10]. And a recent study indicated that physical stress resulted in immediate increase in the plasma concentrations of cortisol [11]. The level of target substances in serum from the upper side of the SST tubes were measured using a BS-400 chemistry analyzer (Mindray, Shenzhen, China) and an Immulite 1000 Immunoassay System (Siemens, New York, USA) [12, 13].

### RNA sequencing and bioinformatic analysis

Total RNA was extracted from the PAXgene tubes according to the manufacturer’s instructions [6]. Starting with the total RNA, mRNA was purified using poly (A) selection or rRNA depletion, converted into double-stranded cDNA, and amplified by PCR. To check the RNA quality, all the RNA samples were examined for RNA Integrity Number, and 28S to 18S rRNA value using Bioanalyzer. Next, the construction of library were used with the Illumina TruSeq RNA Sample Preparation Kit v2 (catalog #RS-122-2001, Illumina, San Diego, CA) following the manufacturer’s instructions [14]. And the library was quantified using the KAPA library quantification kit (Kapa Biosystems KK4854) following the manufacturer’s instructions [15]. The final individual libraries were sequenced using the Illumina Hiseq2000 platform, which created 100 bp paired-end (PE) RNA-sequencing reads.

To collect high-quality transcriptome data, we filtered the sequencing data by phred score (Q ≥ 20) and minimum length (≥25 bp) using the SolexaQA software [16]. The filtered reads were mapped to 48,370 reference mRNAs from *Canis lupus familiaris* using the bowtie2 software (mismatches ≤ 2) [17, 18]. The number of mapped reads for each mRNA was counted and then normalized using the DESeq packages in R [19]. Differentially expressed genes (DEGs) were selected by over 100 mapped read counts, a ≥ twofold change in reads coverage and a binomial test with a false discovery rate (FDR) ≤ 0.01 at the first. And then, the final DEGs were identified by a ≥ twofold change in reads coverage, a binomial test with a false discovery rate (FDR) ≤ 0.01, and a read count ≥ 1,000 either before or after exercise. The FDR was applied to identify the threshold p-value for multiple tests and was calculated using DESeq. Correlation analysis and hierarchical clustering was performed to group the genes according to patterns of expression using the AMAP library in R [20].

And we tested the stability of the gene expression levels before and after the exercise of housekeeping genes, such as *HNRNPH1, GAPDH, RPL8, TAF4B*, and *TAF1* with *t*-test method supporting the statistical significance [21].

### Functional enrichment analysis

Functional enrichment analyses were carried out using the Gene Ontology (GO) database and including all three GO categories (biological processes, cellular components, and molecular functions), providing a structured and controlled vocabulary to describe the gene products [22]. We also used the KEGG database to identify the biological mechanisms and metabolic pathways associated with the differentially expressed genes corresponding their enzyme commission numbers [23]. DAVID is a web-accessible annotation system (https://david.ncifcrf.gov/home.jsp) that provides a comprehensive set of functional annotation tools for investigators for understanding the biological meanings behind large lists of genes [24]. We used DAVID to analyze the clusters of differentially expressed genes annotated by the Entrez gene IDs of the genes with counts ≥ 2 and FDR ≤ 0.1 of each GO and KEGG term.

### Statistical analysis

To identify correlations between the gene expression pattern and body weight, we carried out t-tests comparing W/BH, W/BL, and W/DC values between two groups of dogs that showed different gene expression patterns. To control for group differences in baseline weight, the weight was divided by the BH, BL, and DC. A *p*-value ≤ 0.05 was used as the cutoff for significance in all analyses using the *t*-test function of R.

## Results and discussion

### Morphological traits and blood sample collection

The basic morphological traits of the 10 Sapsarees measured before the experiment are shown in Table 1. All the dogs were males, 13 to 60 months of age. The BH measured from the ground to the top of the withers ranged from 49 cm to 62 cm. The BL measured from the point of the shoulder to the rear point of the croup ranged from 58 cm to 70 cm. The DC measured from the elbow to the top of the withers ranged from 21 cm to 28 cm. The W measured by weighing balance ranged from 18.7 kg to 30 kg [25].

**Table 1:** Summary of the morphological traits of 10 Sapsarees

Group	Name	Birth	Month of age^a^	Sex	Hair color^b^	BH^c^ (cm)	BL^d^ (cm)	DC^e^ (cm)	W^f^ (kg)	W/BH	W/BL	W/CL
I	Cheongbaek	2011.02.15	46	Male	BT	58	65	27	22	0.38	0.34	0.81
I	Rookie	2010.03.23	57	Male	W	62	67	25	24.5	0.40	0.37	0.98
I	Chaeum	2010.10.24	50	Male	Y	50	60	21	15.4	0.31	0.26	0.73
I	Hwangryong	2011.11.08	37	Male	DY	60	69	25	20	0.33	0.29	0.80
I	Pyeonggang	2013.11.19	13	Male	BT	49	59	21	18.7	0.38	0.32	0.89
	Average	-	-	-	-	55.8	64	23.8	20.12	0.36	0.316	0.842
II	Tong	2010.08.15	52	Male	BT	58	66	26	31	0.53	0.47	1.19
II	Huimang	2009.12.11	60	Male	W	58	68	28	24	0.41	0.35	0.86
II	Pyeongtan	2013.11.17	13	Male	Y	54	63	22	25.5	0.47	0.40	1.16
II	Hwangdol	2011.09.29	39	Male	SY	62	70	27	30	0.48	0.43	1.11
II	Bongsik	2010.07.22	53	Male	DY	53	58	23	19	0.36	0.33	0.83
-	Average	-	-	-	-	57	65	25.2	25.9	0.45	0.396	1.03

^a^Months of age measured from birth to the date of exercise

^b^Hair color : *BT* (Black & Tan), *W* (White), *Y* (Yellow), *DY* (Dark Yellow), *SY* (Strong Yellow)

^c^ *BH* Body Height, ^d^ *BL* Body Length, ^e^ *DC* Depth of Chest, ^f^ *W* Body Weight

### Physical stress indicators in serum

Compared with those before exercise, the concentrations of AST, CK, and creatinine were slightly increased after exercise, but the increases were not significant (Additional file 1: Tables S1 and S2). Cortisol, a key hormone from the adrenal glands, was significantly elevated after exercise in all individuals except for Cheongbaek (Additional file 2: Figure S1, Additional file 1: Table S2). Hence, it seems that cortisol can be used as a marker of exercise-induced stress.

### RNA sequencing and Bioinformatic analysis

After the RNA quality check, the RNA sequencing generated over 75 Gbp (about 3.8 Gbp per sample) of data consisting of 100 bp paired-end reads (Table 2). Trimming resulted in reads with a mean length of 86.58 bp across all samples and a total combined length of about 57 Gbp, which was 74.9 % of the raw sequence.

**Table 2:** Summary of raw sequencing reads

Name	Exercise	Num. of reads (ea)	Avg. length (bp)	Total length (bp)
Cheongbaek	before	31533452	100	3153345200
	after	38271980	100	3827198000
Rookie	before	41724150	100	4172415000
	after	38515768	100	3851576800
Chaeum	before	34658490	100	3465849000
	after	33784972	100	3378497200
Hwangryong	before	36358784	100	3635878400
	after	41631454	100	4163145400
Pyeonggang	before	38708814	100	3870881400
	after	43781622	100	4378162200
Tong	before	40314536	100	4031453600
	after	39786602	100	3978660200
Huimang	before	35384558	100	3538455800
	after	32013742	100	3201374200
Pyeongtan	before	41878502	100	4187850200
	after	40729858	100	4072985800
Hwangdol	before	39336568	100	3933656800
	after	38413046	100	3841304600
Bongsik	before	33239988	100	3323998800
	after	37272096	100	3727209600
Total	**-**	75733982	100	75733898200 (100 %)

**Fig. 1:**
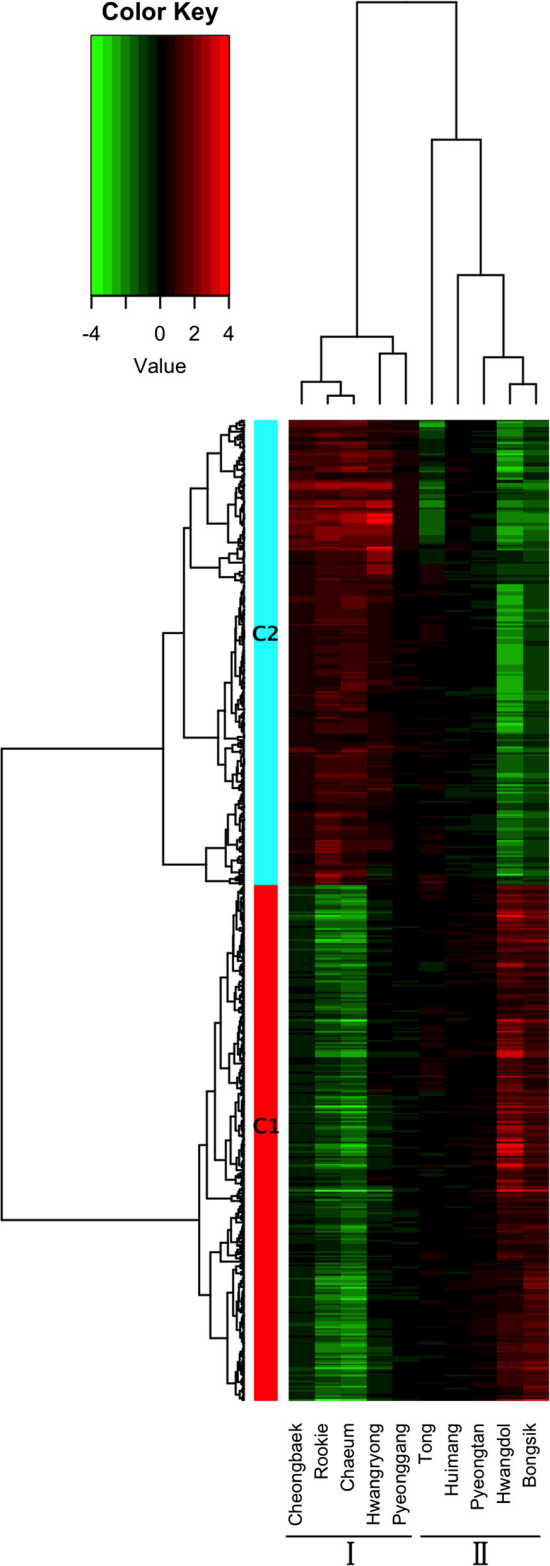
HeatMap showing hierarchical clustering of differentially expressed genes regulated by exercise. The log2Ratio for each significantly differentially expressed gene was used. Each column represents a Sapsaree individual, and each row represents a differentially expressed gene. Expression differences are shown in different colors; red indicates up-regulation after exercise, and green indicates down-regulation after exercise. The 525 genes were grouped into two clusters (C1 and C2). The dogs were divided into two groups. Group I included Cheongbaek, Rookie, Chaeum, Hwangryong, and Pyeonggang. Group II included Tong, Huimang, Pyeongtan, Hwangdol, and Bongsik

Using bowtie2, 80.76 % of the filtered reads were successfully mapped to the current dog reference genes (*Canis lupus familiaris,* 48,370 mRNAs) [18, 26]. A novel bioinformatics pipeline for processing large amounts of transcriptome sequences was built. We calculated the expression levels of all the genes with mapped reads from the 10 individuals before and after exercise. By comparing the coverage before and after exercise, we identified 2,549 DEGs. The numbers of up-regulated and down-regulated genes were different in each individual. Pyeonggang, Huimang, and Pyeongtan had fewer than 10 DEGs, but each of the other dogs had more than 100 genes (Additional file 3: Figure S2). After filtering out genes with low levels of expression, 525 genes were grouped into two clusters (C1 and C2) of 276 and 249 genes, respectively, depending on their expression pattern (Fig. 1 and Additional file 4).

And the reliability of the gene expression analysis based on RNA-Seq was confirmed by *t*-test which supported the statistical significance of the stability of housekeeping genes expression before and after the exercise (Additional file 1: Table S3).

### Functional enrichment analysis

A total of 26 unique genes among the 525 differentially expressed genes were assigned to 11 functional groups based on GO assignments (Additional file 1: Table S4). The genes were involved in biological processes such as programmed cell death, negative regulation of cell motion, regulation of cellular component biogenesis, protein metabolic process, and others. The cellular components linked to the genes were the intracellular parts, such as cytoplasm. The molecular function assignments were mainly to the catalytic and binding activities, such as cation binding.

A further functional classification of all the differentially expressed genes was performed using the KEGG database. A total of 84 unique genes among the 525 differentially expressed genes were assigned to 30 metabolic pathway terms, including phosphatidylinositol signaling system, ribosome, proteasome, oxidative phosphorylation, and others (Additional file 1: Table S5). The functional annotation analyses indicated that the genes regulated under exercise-induced stress were mainly related to the metabolite pathways.

### Correlation between gene expression pattern and body type

Based on the gene expression patterns induced by exercise, the 10 Sapsaree individuals were divided into two groups (Groups I and II; Fig. 1). Group I included Cheongbaek, Rookie, Chaeum, Hwangryong, and Pyeonggang, while Group II included Tong, Huimang, Pyeongtan, Hwangdol, and Bongsik. The differentially expressed genes formed two clusters. Cluster 1 (C1) was down-regulated after exercise in Group I but up-regulated after exercise in Group II. Cluster 2 (C2) was up-regulated after exercise in Group I but down-regulated after exercise in Group II.

We examined the cortisol change after exercise, age, and body type for differences between the two groups. Only the body type showed a significant difference between Group I and Group II. Using W/BH, W/BL, and W/DC to characterize the overall body type, we found that Group I had a heavier body type than Group II (Table 1). In addition, W/BH, W/BL, and W/DC were each significantly different (*p ≤ 0.05*) between the two groups based on independent t-tests (Table 3). Those findings suggest differences in gene regulation between heavier dogs and lighter dogs under exercise stress.

**Table 3:** Differences in body type between groups I and II

	Mean of group I^b^	Mean of group II^c^	t^d^	df^e^	*p*-value^f^
W/BH^a^	0.357	0.452	-3.386	8.124	0.0093
W/BL^a^	0.312	0.397	-3.211	9.413	0.0100
W/DC^a^	0.823	1.018	-2.598	8.364	0.0306

^a^ *W* Body Weight, *BH* Body Height, *BL* Body Length, *DC* Depth of Chest

^b^Mean of Group I: average value of Group I

^c^Mean of Group II: average value of Group II

^d^t : t-value, ^e^df : the degree of freedom value, ^f^ *p*-value : significance

### Identification of candidate marker genes for different response patterns to exercise-induced stress

**Fig. 2:**
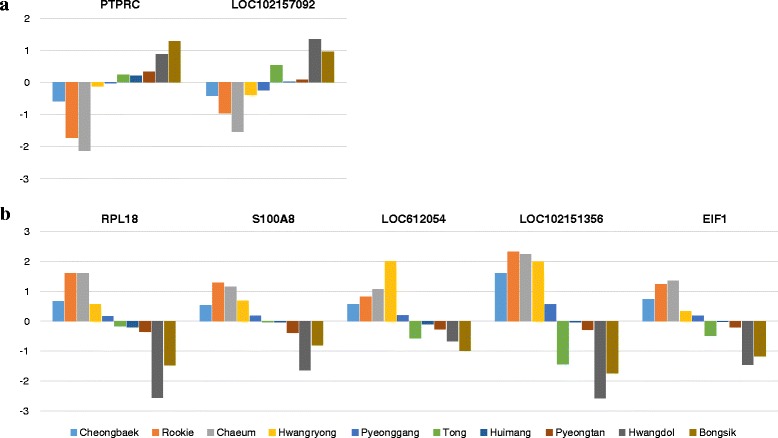
Representative genes that were differentially expressed between the two groups of dogs. The x-axis represents the seven selected genes, and the y-axis represents the log2 fold change (2FC) value of each gene. The 2FC values for each individual are represented by a colored bar. **a** shows two genes with negative 2FC values in Group I and positive 2FC values in Group II. **b** shows five genes with positive 2FC values in Group I and negative 2FC values in Group II. The gene identifiers are shown at the top of each histogram: PTPRC (490255, protein tyrosine phosphatase, receptor type **c**), LOC102157092 (102157092, complement receptor type 1-like), RPL18 (476422, ribosomal protein L18), S100A8 (490461, S100 calcium binding protein A8), LOC612054 (612054, uncharacterized LOC612054), LOC102151356 (102151356, uncharacterized LOC102151356), and EIF1 (403674, eukaryotic translation initiation factor 1)

We identified 525 genes with differential expression before and after exercise. We manually curated seven genes (*PTPRC, LOC102157092, RPL18, S100A8, LOC612054, LOC102151356, EIF1*) that had distinctly different expression patterns before and after exercise in Groups I and II (Fig. 2). For example, *PTPRC* (Entrez ID : *490255*) is described as ‘protein tyrosine phosphatase, receptor type, C’. Some genes had uncharacterized functions. Figure 2 shows the log2 fold changes of the seven selected genes after exercise for each of the dogs. There were clear differences in the changes in expression levels of the seven selected genes after exercise between the two groups of dogs. The seven selected genes could be used as markers of exercise stress and for grouping dogs according to body type.

## Conclusions

This study provides the Sapsaree whole-transcriptome sequence data and identifies genes that are differentially expressed before and after exercise in the Sapsaree, which could be used as markers of exercise stress. The pattern of changes in global gene expression induced by exercise was different depending on the body type. RNA sequencing and gene expression analysis can be useful for grouping the Sapsarees by body type and response to exercise. This study provides a basis for future research investigating the biologic characteristics of the Sapsaree and the large-scale genomic differences of canines in general.

## Additional files

Additional file 1: Table S1. Concentration of four substances in blood at before and after exercise. **Table S2**. The test for statistical significance of the four substances in serum level of before and after exercise. **Table S3**. The test for statistical significance in the stability of housekeeping genes expression before and after exercise. **Table S4**. GO functional classification of DEGs in cluster. **Table S5**. KEGG metabolic pathway annotation of DEGs in cluster. (DOCX 34 kb) Additional file 2: Figure S1. Cortisol concentration change in blood before and after exercise. (PPTX 50 kb) Additional file 3: Figure S2. MAplot showing the expression pattern and the number of genes in each Sapsaree before and after exercise. Expression pattern is shown in different colors dots; red dots indicates up-regulation and green dots indicates down-regulation. And right-top side showing the number of up- regulated and down-regulated genes. Pyeonggang, Huimang, and Pyeongtan had only a few differential gene expressed before and after exercise while others had many more. (PPTX 491 kb) Additional file 4: Gene expression value of the 525 DEGs. (XLSX 163 kb)

## Abbreviations

BH: body height
BL: body length
DC: depth of chest
W: body weight
RNA-Seq: RNA sequencing
DAVID: database for annotation, visualization, and integrated discovery
KEGG: kyoto encyclopedia of genes and genomes
