# Supplementary material for: Whole-transcriptome analyses of the Sapsaree, a Korean natural monument, before and after exercise-induced stress
Source: J Anim Sci Technol. 2016 Apr 15;58:17. doi: 10.1186/s40781-016-0097-1 (PMC4832554; doi:10.1186/s40781-016-0097-1)
Supplement: Additional file 1: Table S1. — Concentration of four substances in blood at before and after exercise. Table S2. The test for statistical significance of the four substances in serum level of before and after exercise. Table S3. The test for statistical significance in the stability of housekeeping genes expression before and after exercise. Table S4. GO functional classification of DEGs in cluster. Table S5. KEGG metabolic pathway annotation of DEGs in cluster. (DOCX 34 kb) [file 40781_2016_97_MOESM1_ESM.docx]

**Additional file 1**

**Table S1. Concentration of four substances in blood at before and after exercise**

| Name | Exercise | AST (u/L) | CK (u/L) | Creatinine (mg/dL) | Cortisol (ug/dL) |
| --- | --- | --- | --- | --- | --- |
| Cheongbaek | before | 30 | 203 | 1.38 | 2.57 |
|  | after | 31 | 194 | 1.45 | 2.55 |
| Rookie | before | 30 | 338 | 1.05 | 2.42 |
|  | after | 31 | 221 | 1.13 | 6.73 |
| Chaeum | before | 27 | 261 | 1.31 | 2.21 |
|  | after | 37 | 364 | 1.43 | 8.99 |
| Hwangryong | before | 23 | 114 | 1.25 | 4.51 |
|  | after | 25 | 147 | 1.34 | 7.97 |
| Pyeonggang | before | 32 | 221 | 1.34 | 6.78 |
|  | after | 37 | 274 | 1.41 | 10.4 |
| Tong | before | 46 | 295 | 1.16 | 2.22 |
|  | after | 42 | 308 | 1.28 | 4.12 |
| Huimang | before | 27 | 156 | 1.14 | 2.89 |
|  | after | 29 | 187 | 1.13 | 4.33 |
| Pyeongtan | before | 29 | 163 | 1.15 | 1.09 |
|  | after | 30 | 198 | 1.29 | 4.9 |
| Hwangdol | before | 26 | 190 | 0.87 | 1.79 |
|  | after | 32 | 418 | 0.92 | 4.5 |
| Bongsik | before | 33 | 334 | 1.24 | 6.34 |
|  | after | 39 | 488 | 1.3 | 7.12 |

*The date of exercise (18-12-2014)

**Table S2. The test for statistical significance of the four substances in serum level of before and after exercise**

|  | Mean of A^a^ | Mean of B^b^ | t^c^ | df^d^ | p-value^e^ |
| --- | --- | --- | --- | --- | --- |
| AST | 33.583 | 31.417 | 0.8926 | 20.929 | 0.3822 |
| CK | 271.750 | 231.417 | 1.1045 | 19.524 | 0.2828 |
| Creatinine | 1.228 | 1.153 | 1.0684 | 21.746 | 0.2971 |
| Cortisol | 5.703 | 3.164 | 2.8566 | 19.896 | **0.0098** |

^a^Mean of A : average value of the hormone concentration before exercise

**^b^**Mean of B : average value of the hormone concentration after exercise

**^c^**t : t-value, **^d^**df : the degree of freedom value, **^e^**p-value : significance

*p ≤*0.05* not likely to be a result of chance (same as saying A ≠ B)

**Table S3. The test for statistical significance in the stability of housekeeping genes expression before and after exercise**

| Gene symbol | Entrez ID | Gene Description | Mean of A^a^ | Mean of B^b^ | t^c^ | df^d^ | p-value^e^ |
| --- | --- | --- | --- | --- | --- | --- | --- |
| HNRNPH1 | 481455 | heterogeneous nuclear ribonucleoprotein H1 (H) | -1.415 | -0.061 | -2.566 | 7.141 | **0.037** |
| LOC485733 (GAPDH) | 485733 | glyceraldehyde-3-phosphate dehydrogenase pseudogene | 0.505 | 2.375 | 3.554 | 7.987 | **0.007** |
| RPL8 | 475130 | ribosomal protein L8 | 0.031 | 2.877 | 2.547 | 5.578 | **0.047** |
| TAF4B | 490507 | TAF4b RNA polymerase II, TATA box binding protein (TBP)-associated factor, 105kDa | 0.326 | 1.598 | 3.730 | 5.814 | **0.010** |
| TAF1 | 491950 | TAF1 RNA polymerase II, TATA box binding protein (TBP)-associated factor, 250kDa | -2.474 | -0.134 | -2.618 | 7.248 | **0.033** |

^a^Mean of A : average value of fold change of gene expression before exercise

**^b^**Mean of B : average value of fold change of gene expression after exercise

**^c^**t : t-value, **^d^**df : the degree of freedom value, **^e^**p-value : significance

*p ≤*0.05* not likely to be a result of chance (same as saying A ≠ B)

**Table S4. GO functional classification of DEGs in cluster**

| Cluster | Category | Accession | Term | Number of genes | p-value |
| --- | --- | --- | --- | --- | --- |
| 1 | BP | GO:0012501 | programmed cell death | 4 | 0.002 |
| 1 | BP | GO:0051271 | negative regulation of cell motion | 2 | 0.040 |
| 1 | BP | GO:0044087 | regulation of cellular component biogenesis | 2 | 0.049 |
| 1 | BP | GO:0010941 | regulation of cell death | 3 | 0.059 |
| 1 | BP | GO:0019538 | protein metabolic process | 4 | 0.087 |
| 1 | BP | GO:0051270 | regulation of cell motion | 2 | 0.097 |
| 2 | CC | GO:0005737 | cytoplasm | 11 | 0.008 |
| 2 | CC | GO:0044424 | intracellular part | 11 | 0.048 |
| 2 | CC | GO:0005622 | intracellular | 11 | 0.058 |
| 2 | CC | GO:0005840 | ribosome | 2 | 0.069 |
| 2 | MF | GO:0043169 | cation binding | 7 | 0.083 |

*GO depth = 3, FDR ≤ 0.1, count ≥ 2

**Table S5. KEGG metabolic pathway annotation of DEGs in cluster**

| Cluster | Accession | Term | Number of genes | p-value |
| --- | --- | --- | --- | --- |
| 1 | cfa04070 | Phosphatidylinositol signaling system | 7 | 0.001 |
| 1 | cfa05213 | Endometrial cancer | 6 | 0.001 |
| 1 | cfa00562 | Inositol phosphate metabolism | 5 | 0.009 |
| 1 | cfa04722 | Neurotrophin signaling pathway | 7 | 0.012 |
| 1 | cfa05215 | Prostate cancer | 6 | 0.014 |
| 1 | cfa04912 | GnRH signaling pathway | 6 | 0.016 |
| 1 | cfa04666 | Fc gamma R-mediated phagocytosis | 6 | 0.018 |
| 1 | cfa05220 | Chronic myeloid leukemia | 5 | 0.025 |
| 1 | cfa04330 | Notch signaling pathway | 4 | 0.028 |
| 1 | cfa04510 | Focal adhesion | 8 | 0.029 |
| 1 | cfa04270 | Vascular smooth muscle contraction | 6 | 0.030 |
| 1 | cfa04810 | Regulation of actin cytoskeleton | 8 | 0.037 |
| 1 | cfa05210 | Colorectal cancer | 5 | 0.041 |
| 1 | cfa04910 | Insulin signaling pathway | 6 | 0.047 |
| 1 | cfa04062 | Chemokine signaling pathway | 7 | 0.047 |
| 1 | cfa04320 | Dorso-ventral axis formation | 3 | 0.050 |
| 1 | cfa04650 | Natural killer cell mediated cytotoxicity | 5 | 0.061 |
| 1 | cfa05200 | Pathways in cancer | 10 | 0.062 |
| 1 | cfa05216 | Thyroid cancer | 3 | 0.080 |
| 1 | cfa04730 | Long-term depression | 4 | 0.084 |
| 1 | cfa04114 | Oocyte meiosis | 5 | 0.086 |
| 1 | cfa04660 | T cell receptor signaling pathway | 5 | 0.089 |
| 2 | cfa03010 | Ribosome | 22 | 0.000 |
| 2 | cfa03050 | Proteasome | 8 | 0.000 |
| 2 | cfa00190 | Oxidative phosphorylation | 10 | 0.000 |
| 2 | cfa05012 | Parkinson's disease | 9 | 0.001 |
| 2 | cfa04260 | Cardiac muscle contraction | 5 | 0.018 |
| 2 | cfa05016 | Huntington's disease | 8 | 0.020 |
| 2 | cfa05010 | Alzheimer's disease | 9 | 0.024 |
| 2 | cfa00860 | Porphyrin and chlorophyll metabolism | 3 | 0.062 |

*FDR ≤ 0.1, count ≥ 2
