# Supplementary material for: Whole-transcriptome analyses of the Sapsaree, a Korean natural monument, before and after exercise-induced stress
Source: J Anim Sci Technol. 2016 Apr 15;58:17. doi: 10.1186/s40781-016-0097-1 (PMC4832554; doi:10.1186/s40781-016-0097-1)
Supplement: Additional file 2: Figure S1. — Cortisol concentration change in blood before and after exercise. (PPTX 50 kb) [file 40781_2016_97_MOESM2_ESM.pptx]

## Slide 1
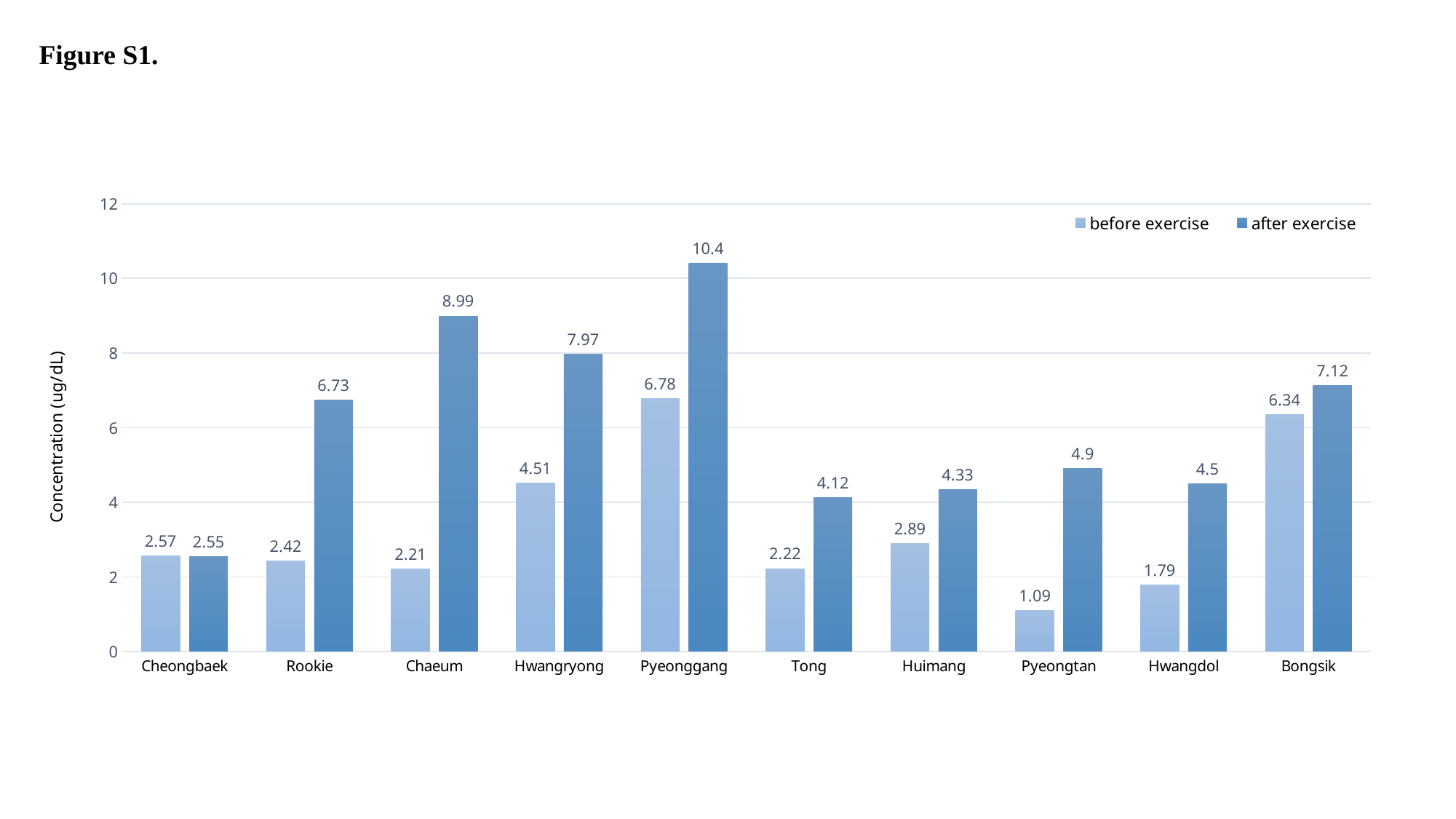

Figure S1.
### Chart
| Category | before exercise | after exercise |
|---|---|---|
| Cheongbaek | 2.57 | 2.55 |
| Rookie | 2.42 | 6.73 |
| Chaeum | 2.21 | 8.99 |
| Hwangryong | 4.51 | 7.97 |
| Pyeonggang | 6.78 | 10.4 |
| Tong | 2.22 | 4.12 |
| Huimang | 2.89 | 4.33 |
| Pyeongtan | 1.09 | 4.9 |
| Hwangdol | 1.79 | 4.5 |
| Bongsik | 6.34 | 7.12 |
