# Supplementary material for: Whole-transcriptome analyses of the Sapsaree, a Korean natural monument, before and after exercise-induced stress
Source: J Anim Sci Technol. 2016 Apr 15;58:17. doi: 10.1186/s40781-016-0097-1 (PMC4832554; doi:10.1186/s40781-016-0097-1)
Supplement: Additional file 3: Figure S2. — MAplot showing the expression pattern and the number of genes in each Sapsaree before and after exercise. Expression pattern is shown in different colors dots; red dots indicates up-regulation and green dots indicates down-regulation. And right-top side showing the number of up- regulated and down-regulated genes. Pyeonggang, Huimang, and Pyeongtan had only a few differential gene expressed before and after exercise while others had many more. (PPTX 491 kb) [file 40781_2016_97_MOESM3_ESM.pptx]

## Slide 1
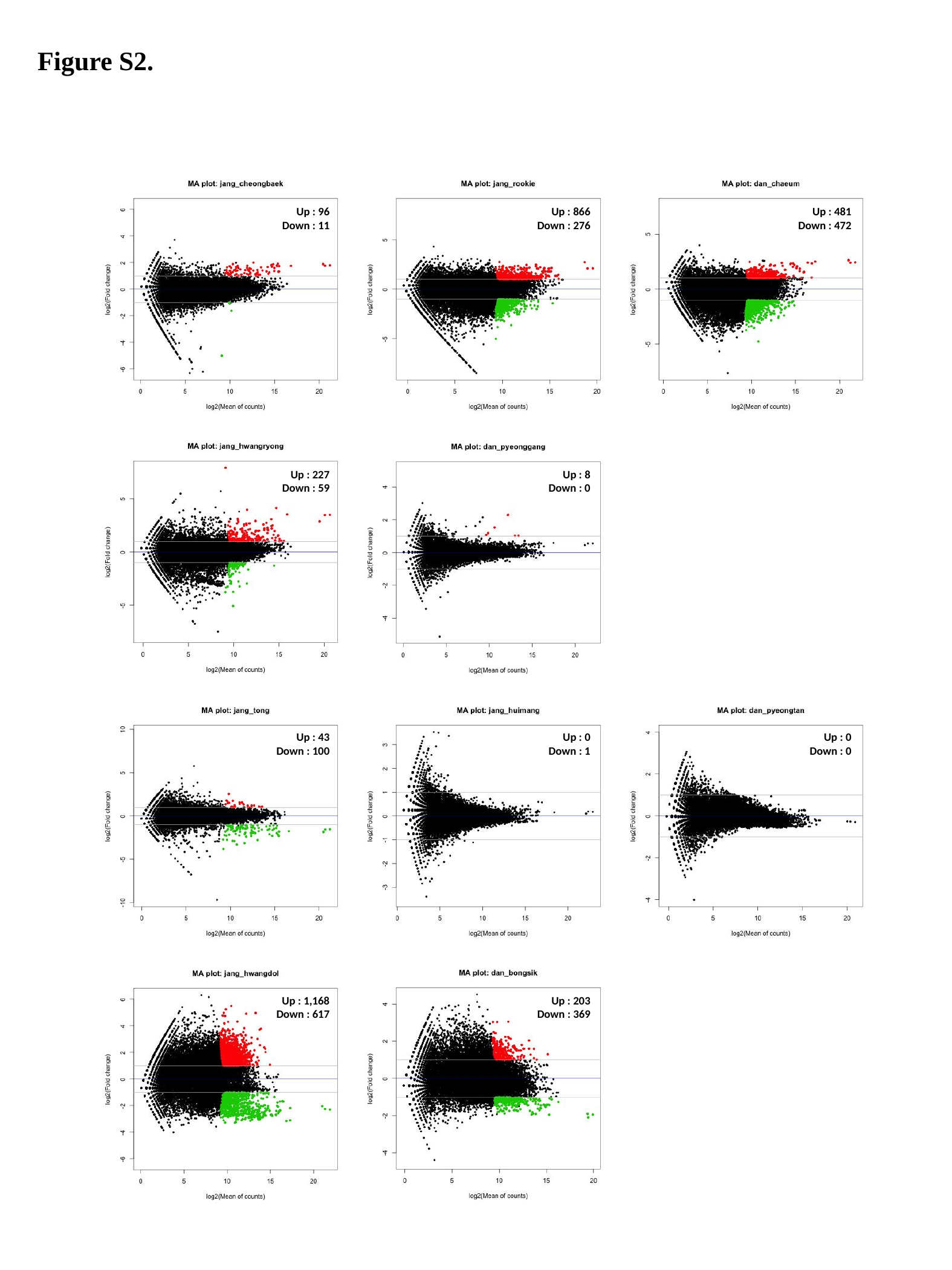

Figure S2.
2549 DEG
기준 FC 2
FDR 0.01이하
Counts 1000 이상
Up : 96
Down : 11
Up : 866
Down : 276
Up : 481
Down : 472
Up : 227
Down : 59
Up : 8
Down : 0
Up : 43
Down : 100
Up : 0
Down : 1
Up : 0
Down : 0
Up : 1,168
Down : 617
Up : 203
Down : 369
